# Conservative Dentistry

**Published:** 1908-11-14

**Authors:** 


### Dental Department.

### CONSERVATIVE DENTISTRY.

Together with other branches of medicine, dental surgery has made enormous strides during the last twenty years. When artificial teeth first came into general use—that is, when the cost ceased to be prohibitive to the ordinary individual, which was about coincident with the discovery of the vulcanite process—they were thought to be such perfectly satisfactory substitutes for Nature's own that the most trifling cavity or irregularity in a tooth sufficed for the dentist to advise its extraction, the smallest amount of pain or discomfort was sufficient for the patient to wish to be quit of it.

### The Modern Point of View.

The conscientious dentist to-day looks upon the masticatory apparatus as a whole; he points out to his patients that it is a mechanical device, perfect for the work it has to do, that its mechanical efficiency depends upon the integrity of every one of its component parts, and that his general well-being depends more than he realises on his maintaining that mechanical efficiency. He looks upon the loss of even one tooth as a disaster, as an essential part missing from the machine, and he maintains that the loss of a single tooth, from caries alone, is unnecessary, and is due to preventable causes.

With the knowledge we have acquired, with the skill we should possess, and with all the mechanical devices at our command, it ought to be possible to carry our patients well on past middle life with a masticatory apparatus serviceable and intact.

After middle life, when physiological activities begin to slow down, and when approaches the quiescence of old age, we can regard the loss of teeth with less misgiving, for then Nature herself acknowledges that they are not so essential; for atrophy of the peridental membrane and shrinkage of the gums, with consequent loosening and loss of the teeth, are among the first of tissue changes which herald old age.

### Co-operation of the Patient.

If we are to carry out the conservative treatment of dental caries to the extent indicated above, it is essential that we have the most thorough appreciation and co-operation of our patients. It is curious to note how slow the general public have been to appreciate conservative dentistry, with what prejudice the man in the street approaches even to-day the idea of having a tooth treated and filled. To reach the ideal in conservative dentistry we have to start with quite young children, for so careless has been the public, so remiss the dentist, that it is quite unusual to come across an adult who has not one or more teeth missing.

We start with the temporary dentition; we examine the child's mouth three or four times a year; we discover cavities when quite small and fill them with the same care and permanency that we give to the second dentition; we retain them in position till the absorption of the root and the *vis a tergo* of the erupting permanent teeth dislodge them.

It is necessary to watch very carefully the erupting second dentition, so that any malposition or malocclusion may be prevented; and by examining the child three times a year toothache can be prevented absolutely, and the loss of teeth guarded against; and when adult life is reached the individual has acquired the habit of attending to his teeth, realising their supreme importance.

It rests with parents to co-operate with the dentist to bring about so desirable a state of things, for without the co-operation of the parent, the dentist can do nothing; and surely it is the duty of the medical practitioner to urge upon parents the importance of keeping the dental mechanism intact?

Thorough conservative dentistry is not practised in England to the extent it is in America, nor has it met with the same appreciation; it is the Englishman's characteristic to muddle along; he responds only to a violent stimulus, and he is impatient of treatment. The extraction of teeth is so easy, and the adaptation of artificial teeth by means of plates seems so satisfactory; it demands so little of the operator's time, and so little endurance on the part of the patient, that only too readily do both agree on this line of treatment.

### The Folly of Premature Extraction.

No one can deny that a perfect set of natural teeth must be better than any kind of artificial substitutes, and a properly filled tooth is as serviceable as one which is in every respect normal, and therefore a set of natural teeth, even if the majority of them are filled, must be better than any artificial substitutes. The points we urge against the premature extraction of teeth are:—

First, that though it is the easiest it is not the best line of treatment; that it leads patients to undervalue their natural teeth and to become careless in their attention to them. Also that artificial teeth have only a fraction of the mechanical efficiency of natural teeth, and that this premature extraction brings about tissue changes, absorption of the alveolus of the maxillæ, shrinkage of the gums, atrophy of the muscles of mastication, which only normally come about with old age. Finally, it leads to adverse changes in the appearance of the individual, adding years to her age, altering her natural expression, and altogether placing her at a distinct disadvantage.

Whilst the West End of London swarms with "beauty doctors" and ladies strain every nerve and spend fabulous sums in the endeavour to keep the semblance of youth, it is strange that they have not realised how the loss of teeth adds years to their age, and how totally inadequate dentures are to compensate for the loss of tissue, to prevent the falling in of the cheeks, with the subsequent flabby appearance of the face, and the development of an unsightly groove above the upper lip.

